# Measurement of Electroretinograms and Visually Evoked Potentials in Awake Moving Mice

**DOI:** 10.1371/journal.pone.0156927

**Published:** 2016-06-03

**Authors:** Yusuke Tomiyama, Kosuke Fujita, Koji M. Nishiguchi, Naoyuki Tokashiki, Reiko Daigaku, Kitako Tabata, Eriko Sugano, Hiroshi Tomita, Toru Nakazawa

**Affiliations:** 1 Department of Ophthalmology, Tohoku University Graduate School of Medicine, Sendai, Japan; 2 Department of Retinal Disease Control, Tohoku University Graduate School of Medicine, Sendai, Japan; 3 Department of Advanced Ophthalmic Medicine, Tohoku University Graduate School of Medicine, Sendai, Japan; 4 Laboratory of Visual Neuroscience, Department of Chemistry and Bioengineering, Faculty of Engineering, Graduate School of Engineering, Iwate University, Morioka, Japan; Eye Hospital, Charité, GERMANY

## Abstract

The development of new treatments for intractable retinal diseases requires reliable functional assessment tools for animal models. *In vivo* measurements of neural activity within visual pathways, including electroretinogram (ERG) and visually evoked potential (VEP) recordings, are commonly used for such purposes. In mice, the ERG and VEPs are usually recorded under general anesthesia, a state that may alter sensory transduction and neurotransmission, but seldom in awake freely moving mice. Therefore, it remains unknown whether the electrophysiological assessment of anesthetized mice accurately reflects the physiological function of the visual pathway. Herein, we describe a novel method to record the ERG and VEPs simultaneously in freely moving mice by immobilizing the head using a custom-built restraining device and placing a rotatable cylinder underneath to allow free running or walking during recording. Injection of the commonly used anesthetic mixture xylazine plus ketamine increased and delayed ERG oscillatory potentials by an average of 67.5% and 36.3%, respectively, compared to unanesthetized mice, while having minimal effects on the a-wave and b-wave. Similarly, components of the VEP were enhanced and delayed by up to 300.2% and 39.3%, respectively, in anesthetized mice. Our method for electrophysiological recording in conscious mice is a sensitive and robust means to assess visual function. It uses a conventional electrophysiological recording system and a simple platform that can be built in any laboratory at low cost. Measurements using this method provide objective indices of mouse visual function with high precision and stability, unaffected by anesthetics.

## Introduction

The electroretinogram (ERG) and visually evoked potentials (VEPs) are standard electrophysiological measures used to assess the *in vivo* function of visual pathways. Current systems allow for long-term and non-invasive recordings in both humans and animals. The key strength of the ERG is the ability to isolate signals from different classes of retinal neurons by changing the light adaption status, stimulus conditions, and data processing parameters. For example, presenting dim flashes after a sufficient period of dark adaptation enables extraction of light responses specifically from rod pathways, whereas recording after light adaptation enables the recording of responses exclusively from cone pathways. Waveform analysis is another means of distinguishing the activities of different retinal cell types by ERG recording. The first negative deflection, termed the “a-wave,” derives mainly from the primary retinal neurons, the photoreceptors, whereas the following positive peak, designated the “b-wave,” mainly reflects responses from the downstream bipolar neurons [1]. Furthermore, within the ERG elicited in response to brighter light flashes, distinct high-frequency wavelets called oscillatory potentials (OPs) are superimposed on the ascending slope of the b-wave. These are believed to reflect inner retinal activity, although their cellular origins are thought to be complex [1, 2]. Nevertheless, because of their unique functional implications, OPs have been used as indices of inner retinal function in animals, including mice. While the ERG reflects retinal activity, VEPS reflect neural activity in the visual cortex; thus, VEP in combination with ERG assessment provides a comprehensive view of the functional integrity of the retinothalmocortical visual pathway.

The ERG and VEPs are usually recorded in animals under general anesthesia because these signals are easily contaminated by electrical noise arising from movements of the body or recording electrodes. A well-tolerated and commonly used anesthetic for *in vivo* electrophysiological recording is a mixture of ketamine and xylazine. However, application of these anesthetics induces various physiological changes in animals, including depression of cardiac function [3, 4] and cerebral hemodynamics [5], reduced body temperature [6], and development of cataracts [7], all of which could affect the cellular responses underlying ERG and VEP signals. Therefore, the ERG and VEPs should be recorded in the conscious state to eliminate spurious drug-related effects. Visually evoked potentials have been recorded from awake animals, including mice [8, 9]. Awake ERG recordings have also been achieved in animals, including rats [10–13], cats [14], and dogs[15], but there are no reports of awake ERG recordings in mice, the laboratory animal that meets most of the practical needs for eye research.

For this study, we developed a simple and inexpensive new platform for recording ERG and VEP in a conscious freely moving mouse. Results revealed that electrophysiological measurements under general anesthesia do not accurately reflect the physiological light responses of the visual pathway, highlighting the importance of visual assessment in awake animals.

## Materials and Methods

### Animals

This study used adult (5–11-week-old) C57BL/6J mice purchased from SLC Inc. (Hamamatsu, Japan). All mice were handled and maintained in accordance with the ARVO Statement guidelines for the Use of Animals in Ophthalmic and Vision Research, and the Declaration of Helsinki, and the intramural Guidelines for the Care and Use of Animals. All experimental procedures were conducted after approval by the Ethics Committee for Animal Experiments at Tohoku University Graduate School of Medicine. Cervical dislocation was applied to sacrifice animals when necessary.

### Head fixation device and recording platform

**Fig 1:**
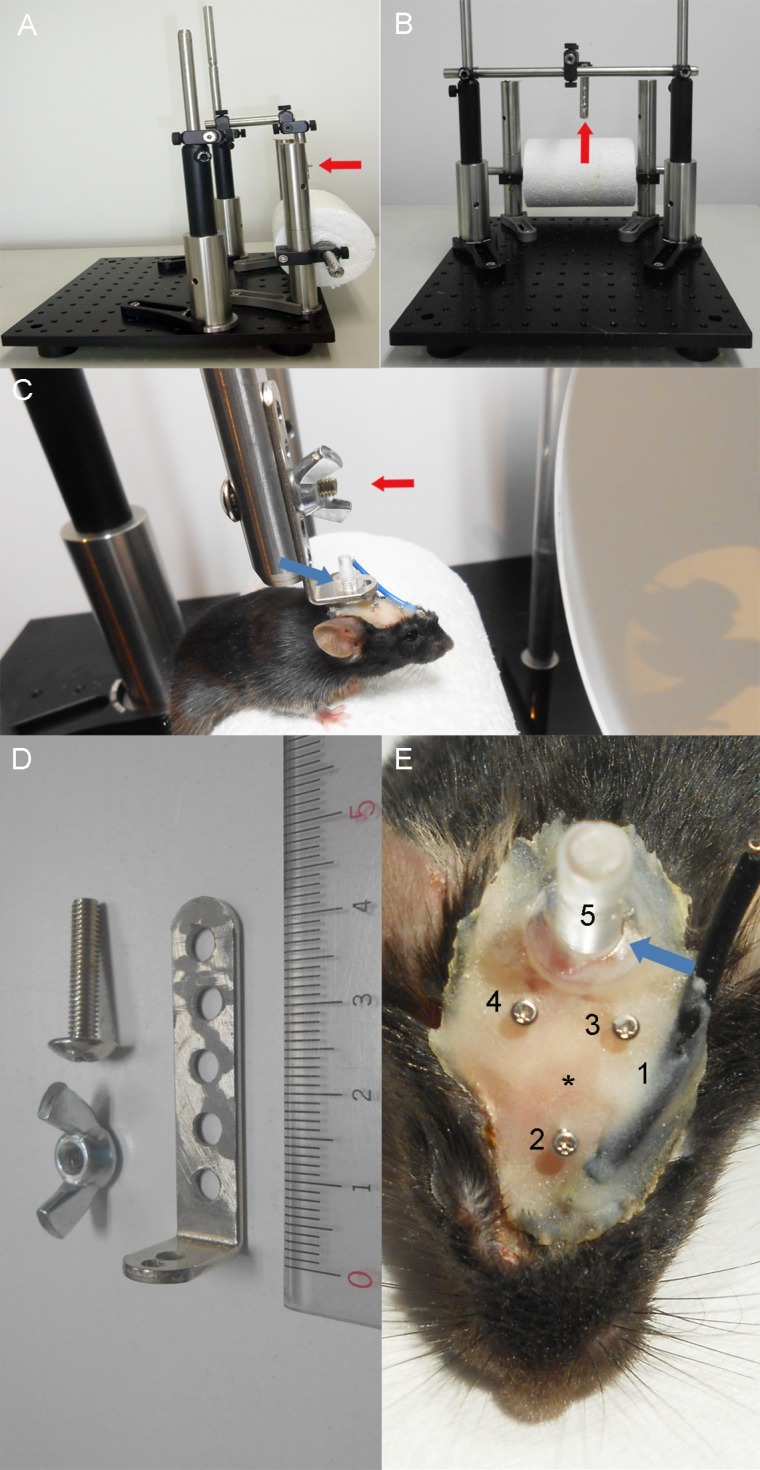
Setup for awake ERG/VEP recording. A, B, Images of the device used to fix the skull of a mouse while recording awake ERGs and VEPs. C, Experimental setup for recording. A mouse with head fixed to the restraint device was placed on a rotatable polystyrene cylinder. The setup allows the mouse to run or walk freely during ERG/VEP recording. Electrical cords, which are not shown in the image, were connected to the electrodes on the skull during recording. D, An adaptor, a nut, and a bolt used to connect a mouse to the vertical attachment of the restraint device (red arrows in panels A, B, and C). The ends of the adaptor linked the mouse skull to the restraint device and platform. E, Screw electrode, fixation bolt, and wire electrode implants: 1, reference wire electrode for ERG; 2, reference screw electrode for VEP; 3, 4, positive screw electrodes for VEP; 5, plastic bolt used to attach the skull to the fixation device (see blue arrows in panels C and E); asterisk, bregma.

The head-restraining device and recording platform are shown in Fig 1A–1C. The device was built in-house and consisted of a head restrainer and a rotating polystyrene foam cylinder fixed on a stable platform. An adjustable adaptor was used to fix the mouse skull to the head restrainer by fastening a plastic bolt implanted on the skull with a nut as displayed in Fig 1C and 1D.

### Surgical preparation of electrodes

Mice were anesthetized using a single intraperitoneal injection of medetomidine (0.6 mg/kg; Meiji Seika Pharma Co. Ltd., Tokyo, Japan) and ketamine (36 mg/kg; Daiichi Sankyo Co. Ltd., Tokyo, Japan) before surgical procedures. A summary of the surgical outcomes is displayed in Fig 1E.

Before implanting VEP electrodes, a part of the scalp around bregma (10.0 mm caudal, 10.0 mm rostral, and 5.0 mm lateral; Fig 1E) was shaved and disinfected using povidone iodine (Meiji Seika Pharma Co. Ltd.). After excising the disinfected skin, subdermal connective tissue was removed from the bone using a scalpel. Electrodes for VEP recordings were inserted over the right and left primary visual cortex (3.6 mm caudal and 2.3 mm lateral to bregma; electrodes 3 and 4 in Fig 1E). The negative electrode was placed over an area of the right prefrontal cortex (2.0 mm rostral to bregma; 2 in Fig 1E). Three stainless steel pan-head screws (M0.6 × 3.0 mm) used for electrodes were inserted 1.0 mm into the skull so that the tips made light contact with the cortical surface. These screws were then fixed in place using cyanoacrylate adhesive (Toagosei Co. Ltd., Tokyo, Japan).

The negative electrode for ERG recording was also surgically implanted. The end of a copper wire (25 mm) was exposed 3 mm on each side. One end of the wire was placed between the two eyes (the black cord in Fig 1E) and fixed to the skull using cyanoacrylate adhesive. The other end was eventually attached to the recording system.

A polycarbonate bolt (M3 × 10.0 mm) was also glued onto the exposed skull caudal to the electrodes to fix the mouse head to the head restraining device. Dental cement (GC Unifast III; GC Dental Products Corp., Tokyo, JAPAN) was applied over the exposed skull to seal the wound. The tops of the stainless screws and the polycarbonate bolt remained free from the adhesive glue and cement. Mice were kept warm on a heating pad during the procedures. After surgery, mice were administered a reversal agent, atipamezole (α2 adrenergic receptor antagonist; 0.35 mg/kg; Meiji Seika Pharma Co. Ltd.).

### Simultaneous recording of ERG and VEP

The ERG and VEPs were recorded using a Ganzfeld dome, an acquisition system (PuREC), and an LED stimulator (LS-100; all from Mayo Corp., Inazawa, Japan). After mice were dark-adapted for at least 6 h, pupils were dilated using 2.5% phenylephrine and 1.0% tropicamide eye drops. Previous studies demonstrated that mouse rod pigment regeneration and dark adaptation are typically complete within 1 h [16, 17], so the minimum 6-h period allowed for full dark adaptation before scotopic experiments. Then, the head of the mouse was firmly fixed to the head restraining device using an adaptor and two pairs of bolts and nuts, while the mouse was allowed to run freely on the rotatable polystyrene cylinder (Fig 1C). Then, anesthetic eye drops (oxybuprocaine hydrochloride; Santen Pharmaceutical Co. Ltd., Osaka, Japan) and moisturizing eye drops (3% sodium hyaluronate and 4% sodium chondroitin sulfate; Viscoat; Alcon Laboratories, Fort Worth, TX) were instilled, after which a contact lens positive electrode for recording the ERG (Mayo Corp.) was placed on the right eye. The negative electrode placed on the skull and ground electrode placed on the tail were connected to the acquisition system. The positive VEP electrode placed over the left visual cortex and negative electrode over the prefrontal cortex were also connected to the acquisition system. The mouse and the device were moved carefully in front of the Ganzfeld dome so that the eyes were positioned at the entrance of the sphere. The mouse was kept in the darkness for a few additional minutes until it became calm and ready to undergo the recordings. Although mice resisted during fixation of the skull to the device, they quickly calmed once fixed and free of our hands, thereafter remaining mostly still or slowly walking on the cylinder.

The ERG and VEPs were first recorded under a scotopic condition using a series of white flashes of (in log cd s/m^2^) −7.0 (0.5 Hz, 100 responses averaged), −6.0 (0.5 Hz, 100 responses averaged), −5.0 (0.5 Hz, 50 responses averaged), −4.0 (0.5 Hz, 50 responses averaged), −3.0 (0.5 Hz, 50 responses averaged), −2.0 (0.5 Hz, 50 responses averaged), −1.0 (0.5 Hz, 50 responses averaged), 0.0 (0.1 Hz, 3 responses averaged), 1.0 (0.016 Hz, 3 responses averaged), 1.5 (0.016 Hz, 3 responses averaged), and 2.0 (no averaging). Then the mouse was light-adapted against white background light (30 cd/m^2^) for 10 min. Reliable VEP measurements required the averaging of 50 or 100 responses. This averaging was only possible for dimmer flashes (−7.0, −6.0, −5.0, −4.0, −3.0, −2.0, −1.0 log cd s/m^2^) without changing the scotopic adaptation status. Photopic ERGs and VEPs were then recorded using white flashes of 0.0, 0.5, 1.0, 1.5, and 2.0 log cd s/m^2^. The VEP signals were band-pass filtered between 0.3 and 50 Hz. During the awake ERG recording session, mice were observed to walk or run on the cylinder.

The mouse was immediately dark-adapted for approximately 6 h after detaching the device from the head. After anesthetizing the mouse by an intraperitoneal injection of ketamine (100 mg/kg) and xylazine (10 mg/kg), the head of the mouse was fixed again onto the device. The ERG and VEPs were recorded following a protocol identical to that used for awake recording.

Oscillatory potentials were extracted by applying 75–300 Hz band-pass filtering [18]. The 1^st^, 2^nd^, 3^rd^, and 4^th^ positive peaks detected after applying the filter were defined as wavelets OP_1_, OP_2_, OP_3,_ and OP_4_. Signal parameters measured under awake and anesthetized conditions were compared by paired *t*-tests after confirming that the data followed normal distribution as assessed by Kolmogorov-Smirnov test, using R (http://www.R-project.org.).

## Results

### Development of a platform for simultaneous recording of the ERG and VEPs in awake mice

A new platform was developed to allow simultaneous ERG and VEP recording in awake mice. First, a head fixation device was designed (Fig 1A–1C). This device consisted of two horizontal arches of different height aligned in parallel and built on a stable base. The higher arch with a vertical bar attached downward in the center was used to fix the mouse head (red arrows in Fig 1A–1C). A freely rotatable polystyrene cylinder attached to the lower arch allowed the animal to walk or run freely during recording. A custom-designed L-type steel adaptor was used to connect the mouse head to the head restraining device (Fig 1C). The device was designed so that the eyes of the mouse can be aligned at the entrance of the Ganzfeld dome to record the ERG and VEPs using a conventional electrophysiology recording unit. Mice were generally calm during the recording sessions. Moreover, the blink frequency was not increased by applying the contact lens electrode or presenting bright flashes in awake mice (S1 Fig). Therefore, there was no need to reject any ERG or VEP responses contaminated by blink or movement artifacts.

### Increased OP amplitudes in anesthetized mice

**Fig 2:**
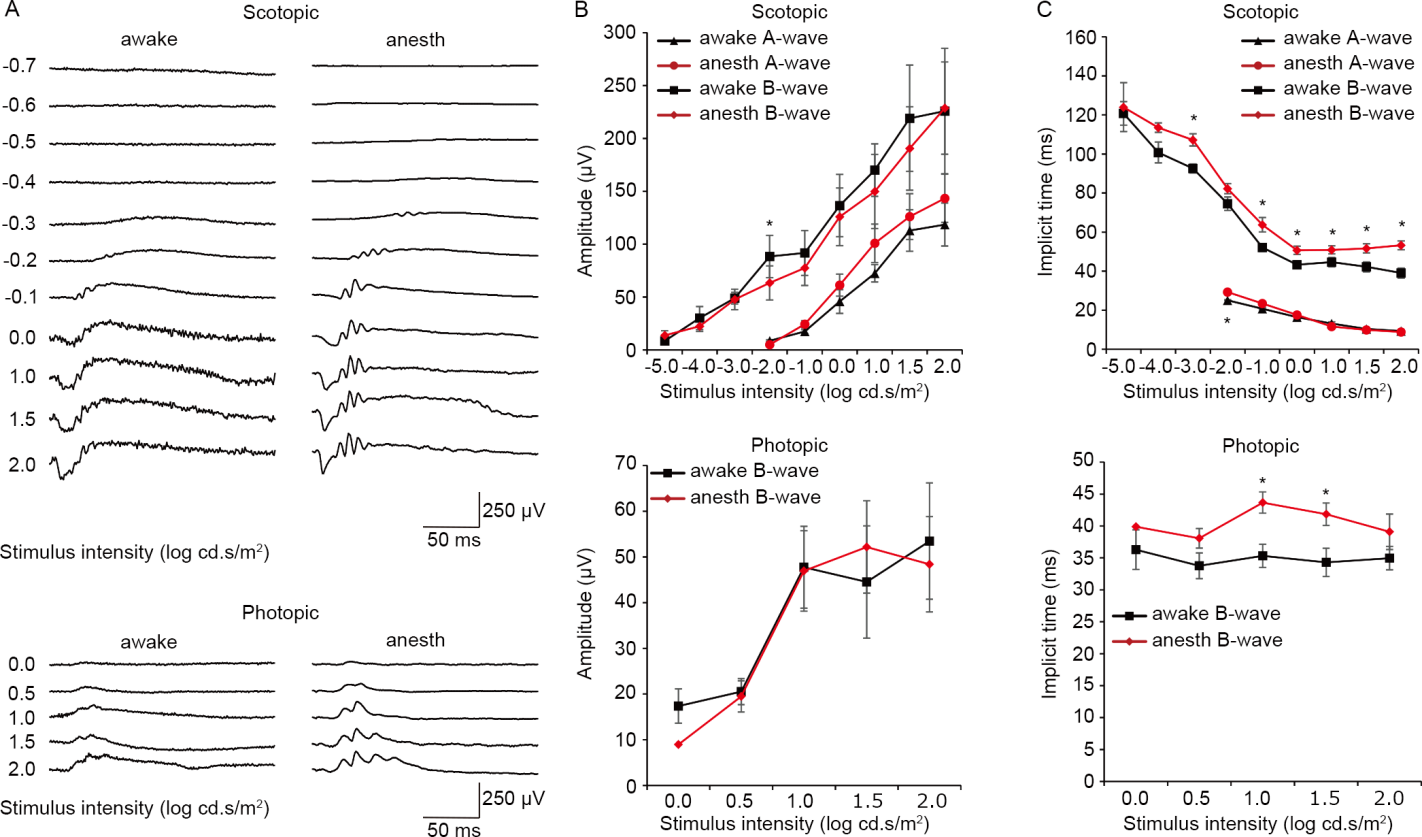
Awake and anesthetized ERG from the same mice. A. Representative traces of scotopic (top) and photopic (bottom) ERGs recorded from the same mouse in the awake (left) and anesthetized (right) condition. B. Amplitudes of scotopic (top) and photopic (bottom) ERG waves from the same mice recorded in awake (black) and anesthetized (red) conditions. C. Implicit times of scotopic (top) and photopic (bottom) ERG waves from the same mice recorded in awake (black) and anesthetized (red) conditions. * *P* < 0.05. Data from seven mice. The bars indicate mean ± standard error of the mean (S.E.M). anes: anesthetized.

Results of the standard scotopic and photopic ERG recordings are presented in Fig 2. The stimulus−response functions acquired during the awake and anesthetized conditions were compared within each mouse to assess anesthetic effects on the a-wave and b-wave. While there were no significant effects of anesthesia on a-wave and b-wave amplitudes, implicit times of b-waves were modestly delayed in anesthetized mice compared to awake mice in both scotopic and photopic trials.

**Fig 3:**
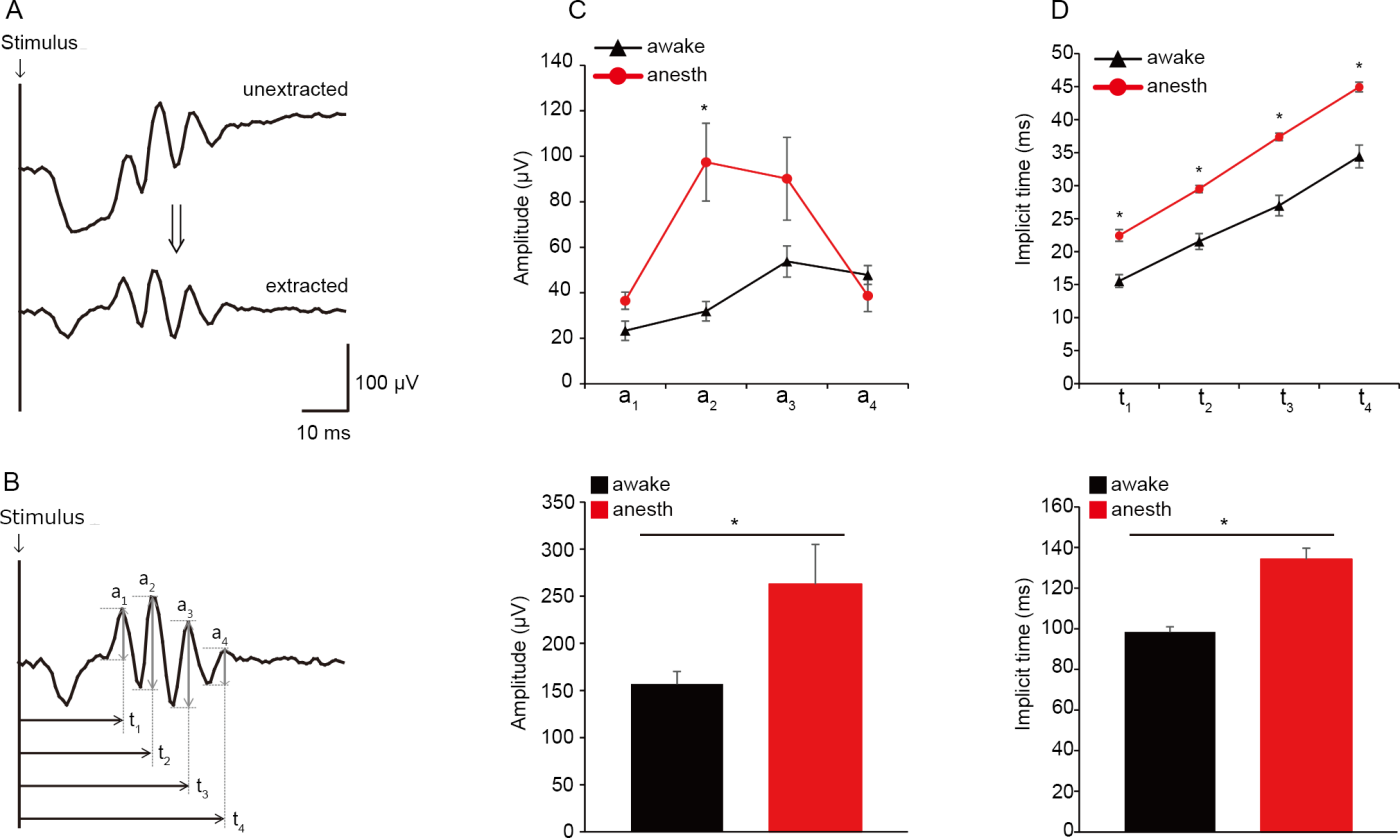
Comparison of OPs extracted from ERGs acquired under awake and anesthetized conditions. Representative ERG traces before and after the extraction of OPs from a scotopic ERG (0 log cd s/m^2^). Schematic illustration showing the amplitude (a_n_) and implicit time (t_n_) of each OP wavelet (OP_n_). C, D. Amplitudes (C) and implicit times (D) of individual OP wavelets (OP_1_–OP_4_) recorded in the awake (black) and anesthetized (red) condition from the same mice. E, F. Summed amplitudes (C) and implicit times (D) of all OP wavelets (OP_1_–OP_4_) from ERGs recorded under awake (black) and anesthetized (red) conditions from the same mice. * *P* < 0.05. Data from seven mice. The bars indicate mean ± S.E.M. anes: anesthetized.

When the ERG traces obtained under awake and anesthetized conditions were compared within mice, gross differences in OPs were noted, particularly in response to flashes of higher intensities. OPs are high-frequency wavelets observed on the ascending slope of the b-wave (Fig 1A) and are regarded as reflecting inner retinal function [1, 2]. Therefore, we compared amplitudes and implicit times of the OPs extracted from scotopic responses to a bright white flash (0 log cd s/m^2^) under awake and anesthetized conditions (Fig 1B). The mean overall OP amplitude, defined as the average sum of amplitudes of all four measured wavelets (OP_1_, OP_2_, OP_3,_ and OP_4_), was substantially larger in anesthetized mice than awake mice (Fig 3C lower panel; 262.7 ± 42.1 μV vs. 156.8 ± 13.2 μV; *P* = 0.048). When individual OP wavelets were analyzed, the amplitude of only the second (OP_2_) wavelet was increased (by 205.1%; *P* = 0.008; Fig 3C upper panel) while the mean amplitudes of the first (OP_1_) and fourth (OP_4_) wavelets were comparable between awake and anesthetized conditions. Mean overall OP implicit time, defined as the average sum of implicit times of all four wavelets, was delayed by 36.3% in anesthetized mice compared to awake mice (*P* = 0.001; Fig 3D lower panel). A small but significant increase in the implicit time of OP_1_ was observed in anesthetized mice (~ 44.3%; *P* = 0.002; Fig 3D upper panel), while the implicit times of the others were similar, indicating that the overall delay was mainly due to the delay of OP_1_.

### Increased but delayed VEPs in anesthetized mice

**Fig 4:**
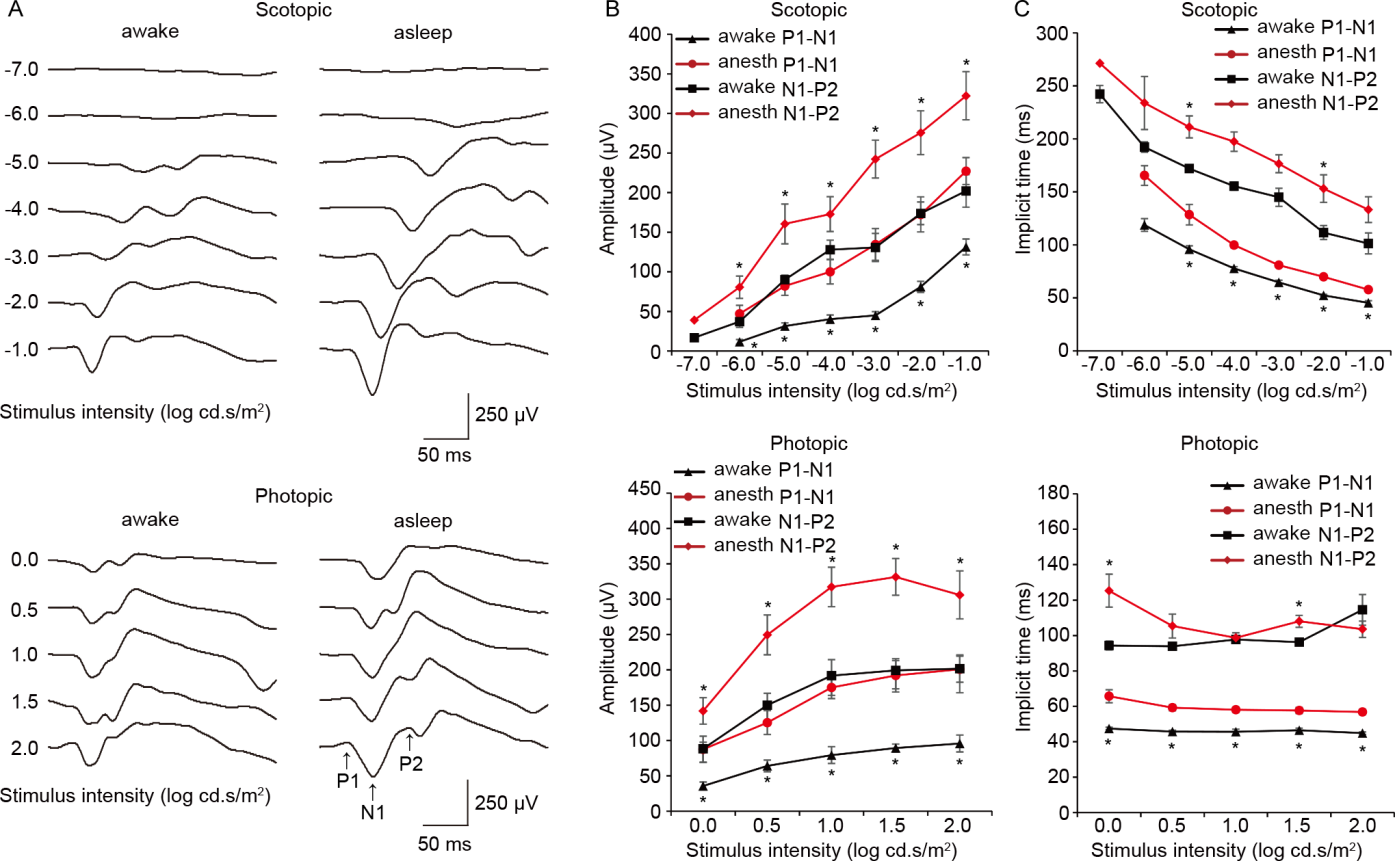
Comparison of VEPs recorded under awake and anesthetized conditions in the same mice. Representative traces of scotopic (top) and photopic (bottom) VEPs from the same mouse recorded in the awake (left) and anesthetized (right) condition. Amplitudes of scotopic (top) and photopic (bottom) VEPs from the same mice recorded in the awake (black) and anesthetized (red) condition. Implicit times of scotopic (top) and photopic (bottom) ERGs from the same mice recorded in the awake (black) and anesthetized (red) condition. * *P* < 0.05. Data from seven mice. The bars indicate mean ± S.E.M. anes: anesthetized.

**Fig 5:**
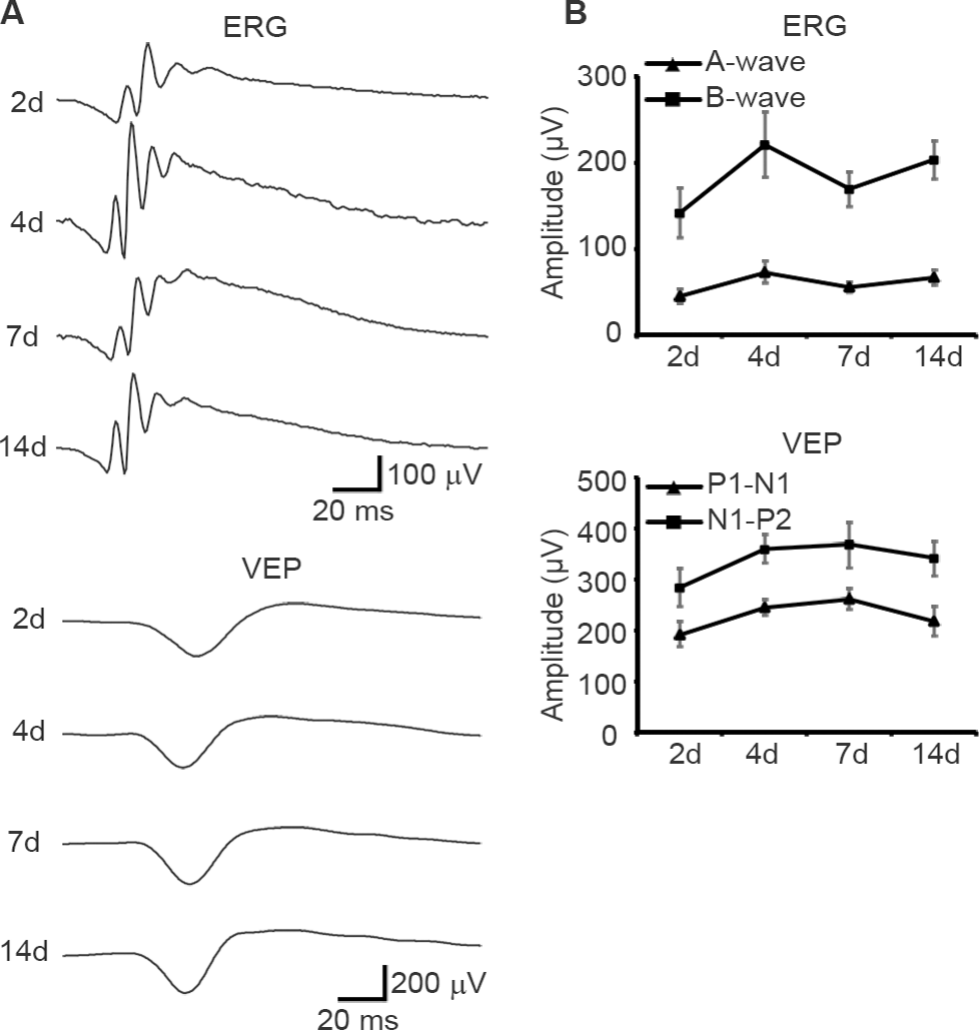
Stability of the ERG and VEP recording. A, B, Amplitude time courses of ERG and VEP waveforms recorded in anesthetized mice (*N* = 4 for each time point). The flashes (0 log cd/m^2^) were given in the scotopic condition. The bars indicate mean ± S.E.M.

Next, we compared flash VEP recordings obtained from the same mice under awake and anesthetized conditions (Fig 4). Both the first negative trough, N1, and the following positive peak, P2, were larger (by up to 302.4% and 130.1%, respectively) but delayed (by up to 39.3% and 37.1%, respectively) in anesthetized mice in both scotopic and photopic trials. The amplitudes of both ERG and VEP waveforms remained relatively stable during 2 weeks of recording (Fig 5). This indicates that conclusions drawn from the current study are valid because ERG and VEP were recorded in both awake and anesthetized conditions from the same mouse on the same day (day 4; see the Methods section for details).

## Discussion

This report describes a new *in vivo* electrophysiology recording platform that enables reliable simultaneous ERG and VEP recording from awake, freely moving mice. This system provided two clear benefits for recording ERG and VEP in awake mice. First, OPs, ERG components believed to originate from the inner retinal cells, were found to be increased and delayed by the anesthetics. Second, VEPs were shown to be enhanced and delayed by the anesthetics.

### New platform for awake ERG/VEP recording in mice

A few groups have reported simultaneous recording of ERG and VEP in awake rats [10–13] and cats [14], but there is no published report characterizing the ERG in awake mice. This is partly because mice are more mobile than rats and mouse eyes are much smaller than those of rats or cats, which together pose a substantial challenge for *in vivo* electrophysiological recording. Furthermore, most earlier reports recorded awake ERGs and VEPs using chronically implanted electrodes [10, 11, 13, 14] without fixing the position of the head and eyes. This may allow the animal to avoid uncomfortable light stimuli, which in turn would affect the light response, ultimately enhancing the variability among trials and animals. To mitigate this risk, we applied complete head fixation during the entire recording so that the eyes were always aligned in the same position, thereby allowing us to deliver consistent light stimuli throughout the experiment. The responses were robust, with minimal noise. No re-recording was necessary because of experimental failure. To relieve some of the stress expected due to head fixation, a freely rotatable cylinder was placed underneath the mouse, which was used by all mice during recording.

Another important feature of the current platform is that it requires no sophisticated devices or tools for construction. Moreover, the surgery is straightforward, taking approximately 15 min per mouse. Consequently, the technique can be replicated easily.

### Comparison of awake and anesthetized ERGs and VEPs

Aside from inconsistent small delays in implicit times under the anesthetized condition, the a-wave and b-wave of the ERG were grossly similar under awake and anesthetized conditions in the same mouse. In contrast, VEP amplitudes were substantially larger in anesthetized mice. The current results are surprisingly similar to those recently reported for rats by Charng et al. [10]. They found only a modest decrease in a-wave and b-wave ERG amplitudes but a substantial increase in flash VEP amplitudes in anesthetized animals. However, the sensitivity of OPs to anesthesia may differ between species. These wavelets were greatly enhanced and delayed in mice, a change not reported in the rat study. Although it is not clear if OPs were analyzed, the ERG waveforms displayed in that paper for the awake and anesthetized conditions appear similar to each other, which suggests that the effect of anesthetics on OPs in rats is slight or negligible. This is in line with a previous report that compared the effects of various anesthetics on rat ERGs and found OPs to be insensitive to the ketamine/xylazine mixture [19]. These results suggest that retinal cells driving the OPs have species-specific pharmacological properties, which must be taken into consideration when interpreting electrophysiology data.

The mechanisms underlying the enhancement of OPs and VEPs by ketamine and xylazine were not explored in the current study. Ketamine acts mainly on N-methyl D-aspartate (NMDA) receptors [20], whereas xylazine is an agonist for α_2_-adrenergic receptors [21], both of which are expressed in the retina and the brain. Neural function in the retina and the brain could be affected directly through these receptors or indirectly through physiological changes, such as lower body temperature [3, 5–7]. Although not analyzed in the current study, isoflurane, another commonly used anesthetic for animal research, reportedly alters the ERG [22] and VEPs [23] in humans. The use of this anesthetic may thus also alter electrophysiological responses in the mouse visual system.

### Implications and limitations

The current study demonstrates that ketamine/xylazine alters both ERG and VEP waveforms. On the ERG, OPs were most dramatically affected, while a-waves and b-waves showed little difference in the presence and absence of anesthetics. These OPs are widely believed to be generated by cells of the inner retina. However, the exact cellular origin is not clear. Neuropharmacological evidence suggests that each OP wavelet arises from a different source. Specifically, they are separable into early, intermediate, and late components generated by photoreceptors, action potential-independent mechanisms, and action potential-dependent mechanisms, respectively, in the ON pathway of the inner retina [1, 2]. This evidence shows agreement with our results that only OP_2_ amplitude was enhanced substantially by the anesthetics, whereas OP_1_ and OP_4_ were clearly unaffected. However, these results should be interpreted with caution, as our study suggests that OPs are especially susceptible to anesthetic effects. Nonetheless, OPs have been studied widely as clinical markers of inner retinal dysfunction in patients with various retinal diseases, including diabetic retinopathy [24], glaucoma [25, 26], vascular occlusions [27], and retinopathy of prematurity [28]. Rodent models of these conditions have shown that OPs are also affected to varying degrees [29–35], but none of the studies analyzed OPs in awake mice. Applying the current technique for measuring OPs in awake animal models of these conditions may provide a more accurate picture of the specific pathogenic mechanisms.

The main limitation of the current approach is the extra time required for surgical preparation, roughly 15 min per mouse. Nevertheless, we believe that the benefits greatly outweigh the increased preparation time for most applications.

## Conclusions

We have developed a new platform for recording ERGs and VEPs in freely moving mice. This platform allows accurate electrophysiological assessment of the functions of the retina and visual cortex. Our methods may have particularly important applications for OP and VEP measurements as these waveforms are strongly modulated by general anesthesia.

## Supporting Information

S1 Fig Quantification of blink reactions during awake ERG recording. Blink frequency measured from the same mouse was not increased by applying contact lens electrode or presenting bright flashes. After placing the mouse in a head-fixation device for awake ERG recording, blink frequency was measured by direct observation of the eyes for 3 minutes using an infrared camera placed inside the Ganzfeld dome with or without contact lens electrode or flashes. Blink frequency was measured first without an electrode (left bar), then with an electrode in the right eye (middle bar), which was followed by exposure to bright flashes without removing the electrode (6 Hz, 2.0 log cd s/m^2^; right bar). Data is expressed as blinks per minute (*N* = 4). The bars indicate mean ± S.E.M.
(TIF)
