# Supplementary figures and images for: Measurement of Electroretinograms and Visually Evoked Potentials in Awake Moving Mice
Source: PLoS One. 2016 Jun 3;11(6):e0156927. doi: 10.1371/journal.pone.0156927 (PMC4892628; doi:10.1371/journal.pone.0156927)

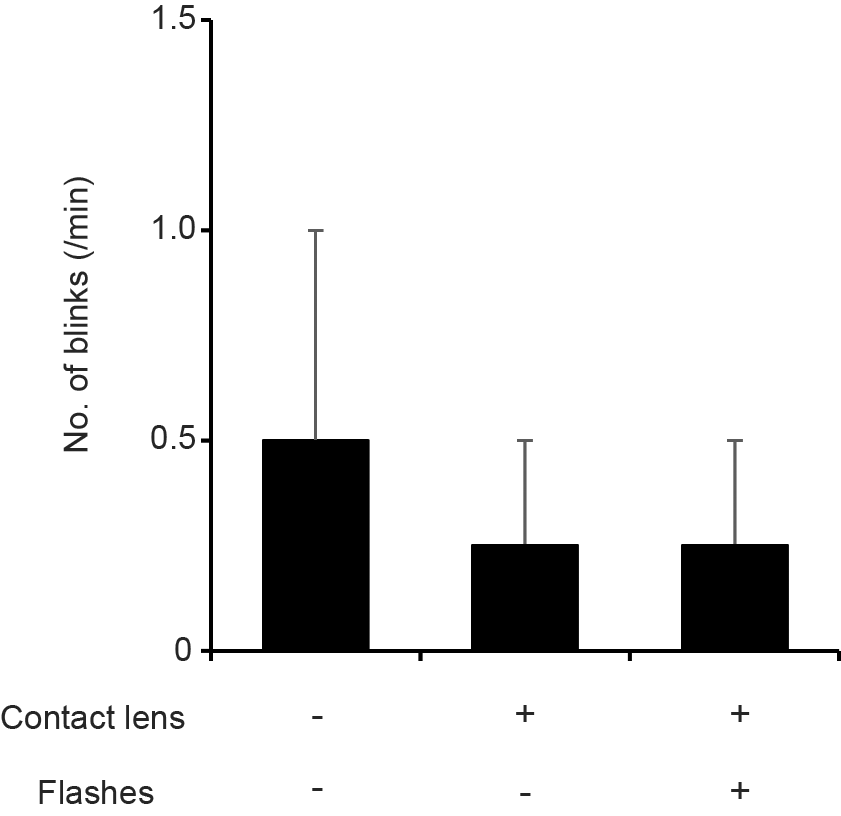

Supplement: S1 Fig — Blink frequency measured from the same mouse was not increased by applying contact lens electrode or presenting bright flashes. After placing the mouse in a head-fixation device for awake ERG recording, blink frequency was measured by direct observation of the eyes for 3 minutes using an infrared camera placed inside the Ganzfeld dome with or without contact lens electrode or flashes. Blink frequency was measured first without an electrode (left bar), then with an electrode in the right eye (middle bar), which was followed by exposure to bright flashes without removing the electrode (6 Hz, 2.0 log cd s/m2; right bar). Data is expressed as blinks per minute (N = 4). The bars indicate mean ± S.E.M. (TIF) [file pone.0156927.s001.tif]
